# Differential expression of chemosensory-protein genes in midguts in response to diet of *Spodoptera litura*

**DOI:** 10.1038/s41598-017-00403-5

**Published:** 2017-03-22

**Authors:** Xin Yi, Jiangwei Qi, Xiaofan Zhou, Mei Ying Hu, Guo Hua Zhong

**Affiliations:** 1 Laboratory of Insect Toxicology, Key Laboratory of Pesticide and Chemical Biology, Ministry of Education, South China Agricultural University, Guangzhou, People’s Republic of China; 2 Guangdong Province Key Laboratory of Microbial Signals and Disease Control, Integrative Microbiology Research Centre, College of Agriculture, South China Agricultural University, Guangzhou, China

## Abstract

While it has been well characterized that chemosensory receptors in guts of mammals have great influence on food preference, much remains elusive in insects. Insect chemosensory proteins (CSPs) are soluble proteins that could deliver chemicals to olfactory and gustatory receptors. Recent studies have identified a number of CSPs expressed in midgut in Lepidoptera insects, which started to reveal their roles in chemical recognition and stimulating appetite in midgut. In this study, we examined expression patterns in midgut of 21 *Spodoptera litura* CSPs (SlitCSPs) characterized from a previously reported transcriptome, and three CSPs were identified to be expressed highly in midgut. The orthologous relationships between midgut expressed CSPs in *S. litura* and those in *Bombyx mori* and *Plutella xylostella* also suggest a conserved pattern of CSP expression in midgut. We further demonstrated that the expression of midgut-CSPs may change in response to different host plants, and SlitCSPs could bind typical chemicals from host plant *in vitro.* Overall, our results suggested midgut expressed SlitCSPs may have functional roles, likely contributing to specialization and adaption to different ecosystems. Better knowledge of this critical component of the chemsensation signaling pathways in midguts may improve our understanding of food preference processes in a new perspective.

## Introduction

The tobacco cutworm, *Spodoptera litura* (F.) (Lepidoptera: Noctuidae), is a generalist herbivore and one of the most important pests in many countries. The economic importance of *S. litura* is owing to its high increase rate and wide host spectrum, encompassing a large assortment of agricultural crops, including vegetables, green manures, and horticultural plants, as well as miscellaneous wild plants and weeds^1^. Chemical cues, emitted from host plants of phytophagous insects, may elicit a wide range of behavioral and physiological responses of insects, including feeding, oviposition and courtship^2^. Among those behaviors, feeding is a complex behavior that could be regulated by several internal mechanisms^3^. Recently, the discovery of chemosensory related proteins in the gut has led to intensive researches on their roles in gut chemical discriminations, stimulating appetite and conditioning food preferences^4^. A myriad of genes in midgut could help maintain balance by prompting foraging and feeding, or by encouraging the cessation of feeding behaviors of insects. Studies suggested that the exposure to plant defense chemicals could have negative effects on insect gut microbial community composition, thus affecting the subsequent feeding behaviors of insects^5^. In addition to this homeostatic regulation, a post-feeding reward system in midgut could also positively reinforce feeding activities^6^. Slight adjustments to midguts system can tilt the balance to affect the nutrient adversity^6^. Extensive studies have been carried out with the upper (oral) regions of chemosensory process to illuminate the mechanisms of host and food preference^7, 8^. However, much remains to be discovered about the lower (gastric, intestinal) regions of the alimentary canal, which were also proved to be critical for the stimulation or inhibition of feeding behaviors^9^.

Normally, the reception of chemical messages in insect starts when chemosensory-related proteins bind the chemicals and transport them through the aqueous hemolymph^10^. Chemosensory protein (CSPs) and odorant binding protein (OBPs) are often speculated to play roles in initial stage of chemosensory perception by insects^11, 12^. While many OBPs have restricted expression pattern in main chemosensory tissues such as antennae, CSPs could express in a variety of tissues and may be involved in divergent functions^13, 14^. One intriguing possibility is that, in midgut, CSPs also could perceive signal chemicals from food resources, thus to mediate feeding behaviors. The analysis of gene expression in response to different treatments have potential to understand the biological function of such genes in adapting surrounding environments^15^ and the binding of an external ligand to the CSPs could result in action potentials and contribute to subsequent behaviors^16^. Eventually, studying of CSPs expression variations in response to different plant resources and binding affinities may elucidate the role of CSPs in midgut.

In our study, 21 CSPs were identified from the transcriptome data we previously conducted. On the quest to challenge our hypothesis, we examined whether the expression levels of selected CSPs in midguts could response to different host plants. And the binding activities between CSPs and typical host plant chemicals were investigated by competitive binding essay *in vitro*. This study could potentially uncover the chemosensory protein variations in midguts in response to host plants, which could surely provide foundation for facilitating the understanding of host recognition and feeding preferences.

## Materials and Methods

### Sample preparation and RNA isolation

*S. litura* (F.) larvae were reared on an artificial diet consisting of soybean, yeast extract, wheat bran, and maintained at 27 °C and 70% RH with a 14: 10 h L: D photoperiod^17^. Adults were transferred to Chinese cabbage [*Brassica campestris* L. ssp. Chinensis (L.)] and raised in a greenhouse at 25 °C and 60–70% RH and the honey was added as a dietary supplement.

The total RNA was extracted using the E.Z.N.A.^TM^ total RNA isolation system kit (Omega, USA) according to the manufacturer’s instructions. The concentration of isolated RNA was examined by Nanophotometer. All tissues were stored at −80 °C until to be used experimentally. One μg of the isolated RNA was transcribed to first-strand cDNA by M-MLV reverse transcriptase (TaKaRa, China) and oligo(dT)_18_ as primer at 42 °C for 60 min. The reaction was terminated by heating at 95 °C for 5 min, and the products were stored at −20 °C.

### Identification of CSPs from transcriptome and genome datasets

CSP genes in *S. litura* were identified from the *de novo* transcriptome assembly previously reported by our group which was based on mixed RNA samples from multiple developmental stages, including larva^18^. The original study was centered on Cry toxin receptor, and to our knowledge, this transcriptome dataset has not been used for any CSP related study. Likely coding regions in the transcriptome assembly were annotated using TransDecoder v2.0.1 and translated protein sequences were checked for the presence of the characteristic domain of CSPs (IPR005055) using InterProScan v5. Protein sequences of CSPs in *S. litura* (identified in the previous step) were used as queries to perform TBLASTN search to identify putative CSP coding regions. For such region, homology-based gene prediction was performed using GeneWise v2.2.0 with the most similar query sequence as reference. All predicted genes were further examined for the presence of the characteristic CSP domain in their translated protein sequences. CSPs in *Bombyx mori* and *Plutella xylostella* were identified from their genome assemblies (downloaded from the LepBase: http://ensembl.lepbase.org/) using the same approach. The identified CSPs in *S. litura* and *P. xylostella* were named after orthologous genes in *B. mori*.

### CSPs expressions in midguts

For each sample, the midguts from five *S. litura* (fourth-instar larva of *S. litura*) were dissected and immediately transferred into eppendorf tubes immersed in liquid nitrogen, and all treatments were conducted in three replicates. The expression levels of all identified SlitCSPs in the midguts of *S. litura* were examined by PCR. The primers of all 21 identified SlitCSPs were designed and synthesized as recommended, and the actin gene of *S. litura* was used to normalize the target gene expression (Table 1). The concentration of the primes used in the reaction is 10 nmol. Amplification was performed by denaturing at 94 °C for 5 min, followed by 27 cycles of 94 °C for 30 s, 60 °C for 30 s and 72 °C for 45 s, with a final extension at 72 °C for 10 min. PCR products were analyzed on 1.2% agarose gels.

**Table 1:** The primers used to carry out the RT-PCR.

Primers	Primer sequence(5′-3′)	Primers	Primer sequence(5′-3′)
SlitCSP11 Forward	GACTGCTGACTGCGTACGGTC	SlitCSP14 Reverse	CCTTGGCATCAGGTGTACAC
SlitCSP11 Reverse	CTTCTTGACCAGGTCCTGCC	SlitCSP15 Forward	CTGTGTGTGCTGACGGTGG
SlitCSP3 Forward	CTGTCGTGCTTGGTCGTGGT	SlitCSP15 Reverse	GGTTCGCTCTTGGACCTG
SlitCSP3 Reverse	ATCCTTCGGGCGTGCAAC	SlitCSP9 Forward	CATCTTGGCGTTGGTGGC
SlitCSP8 Forward	CTACGTCAAGTGCATCCTCGA	SlitCSP9 Reverse	TGTCGTTGTGGCTTCAGGG
SlitCSP8 Reverse	GATCAAGTACTCGATCACACGC	SlitCSP17 Forward	CGATAAGAGCACGATGCAGC
SlitCSP5 Forward	TGTTCGGTCTGGCTGCGGT	SlitCSP17 Reverse	GGTAGTTGCGTTGTACGAAGG
SlitCSP5 Reverse	CACTGCGCTGAGCATCGG	SlitCSP13 Forward	GTACGAGAATGCCAACGACA
SlitCSP12.1 Forward	CCTCGTGTTGTCGATTGTGG	SlitCSP13 Reverse	CCAGTTGCTTCCAGATGTCG
SlitCSP12.1 Reverse	TCCTGGCACCCTTCTTCTG	SlitCSP18 Forward	CATAGCGGTGGTGGACG
SlitCSP12.2 Forward	CCTTAGCTGCTCCACTTGCC	SlitCSP18 Reverse	CAGCAGCAGGCTCGTTG
SlitCSP12.2 Reverse	CGCAAGCTGTGGCTATCAC	SlitCSP2 Forward	ATCGACGCCGTGGTAGCTG
SlitCSP6 Forward	TGGAAAGGACCCTGTACACC	SlitCSP2 Reverse	TGCTTCTGGGCATCCGTACA
SlitCSP6 Reverse	CCCTTGAAAGCGTTGAACG	SlitCSP26 Forward	GACGCCCTATTCGCTGATGA
SlitCSP18 Forward	CCTCACTGCCTACGTCAACTG	SlitCSP26 Reverse	TTTCGCCTTCTGGGATGGAG
SlitCSP18 Reverse	GCCAGGTGTCCAGCTCGTT	SlitCSP10 Forward	TCCTGACGAATGGTCCAAGC
SlitCSP19 Forward	ATGTGTGGTGGCAGTGGC	SlitCSP10 Reverse	GGTACACGTGGTGGTGCTAA
SlitCSP19 Reverse	TGACCTTGTCCGAGCTCTTC	SlitCSP25 Forward	GCCTGGGTTCGTGAGAGAAA
SlitCSP1 Forward	GGTGGCAGCCGACTTCTAC	SlitCSP25 Reverse	GCCATCATGGACGCAATCAC
SlitCSP1 Reverse	TGGACTGCATTTACCACACG	SlitCSP4 Forward	CCGTCTGCTGACTGGGTATG
SlitCSP14 Forward	GTCGTGTTCCTCGTGTGTGT	SlitCSP4 Reverse	CGCTGTCGCTCAGTACACTT
Actin Forward	GCCAACAGGGAGAAGATG	Actin Reverse	CGGTGGTGGTGAAAGAGTA

To measure the midgut expression levels of *B. mori* and *P. xylostella*, the following larval midgut transcriptome data were downloaded from the NCBI Sequence Read Archive database: *B. mori*-SRR1805030, SRR1806712, SRR1806713, SRR1806715, and SRR1806736, and *P. xylostella*-SRR835315, SRR835316, SRR835317. Reads were trimmed for low-quality positions using Trimmomatic v0.35, aligned to respective genome assemblies using STAR v2.4.2a with gene annotations as guidance, and uniquely mapped reads were counted using HTSeq v0.6.1p1. For all genes, the reads per kilobase per million mapped reads (RPKM) values were calculated as a measure of expression level, and the percentile ranks within their respective transcriptomes were determined accordingly.

### Expression pattern of three SlitCSPs in other tissues

The expression patterns of three selected candidate CSPs were further investigated by quantitative real-time PCR (qRT-PCR). RNA samples were isolated from different developmental stages (including first to sixth-instar larvae, pre-pupae, pupae and adult), and different tissues (including cuticle, midguts, fatbody, antennae, heads (without antennae), wings, legs, abdomens, testis and ovary). qRT-PCR was performed using iCycler iQ Real-Time PCR Detection System (Bio-Rad) with SYBR green dye (Taraka, China) binding to double-strand DNA at the end of each elongation cycle. Amplification process was carried out by using the same primers as previously mentioned (Table 1). For all of the tested samples, the concentration of total RNA used in reverse transcription to get first-strand cDNA is 1 μg. All amplifications were performed with three biological replicates. Relative gene expression data were analyzed using the 2^−ΔΔCT^ method as described by Livak^19^.

### Bioassay

As fourth-instar larva of *S. litura* have active feeding behaviors and just enter the first stage of gluttony^20–22^, we used fourth-instar larvae to examine the expression levels of candidate CSPs in midgut. Therefore, fourth-instar larvae from the same piece of egg fraction were collected. The starvation group was set as negative control, which fed on nothing, while the group reared on artificial diet was set as positive control. The other two groups were reared on cabbage (*Brassica camperstris ssp.pekinens*) and tobacco (*Nicotiana tabacum L.*), respectively. Every group has five larvae, and repeated for three times. After 24 h feeding, the midguts of the tested *S. litura* were dissected, and the remains in the midguts were removed. By qRT-PCR, the expression levels of SlitCSPs in midguts after treatments were examined as described previously.

### Expression of recombinan CSPs

The sequences encoding three mature SlitCSPs with *EcoR I* (*GAATTC*) and *Xho I* (*CTCGAG*) were connected to pET32a (Invitrogen, US) by T4 DNA ligase (Takara, China) at 14 °C, and then transformed to BL21 (DE3) competent cells (Takara, China). The selected positive bacterial colony was then inoculated in liquid LB overnight at 37 °C, then transferred 50 μL overnight bacterial liquid to 50 mL fresh LB (Ampicilin 100 μg/mL) until its OD_600_ reached 0.4–0.6. Isopropyl-D-thiogalactoside (IPTG) (0.6 mmol/L) was added and then incubated at various times at 28 °C. After breaking by sonic oscillator, 30 μg of expression product of protein was examined by SDS-PAGE and Western blot. The recombinant protein was purified by affinity chromatography using HisTrap columns prepacked with Ni Sepharose (GE Healthcare) according to the specifications. After overnight dialysis in Tris-HCL (pH = 7.4), the protein was subjected to the Bovine Enterokinase overnight to remove the His-tag. The purified protein was collected and examined by 12% SDS–PAGE. Bradford method was used to determine protein concentration^23^. Purified recombinant SlitCSPs protein was used to immunize rabbit as described previously. The sera of the immunized rabbit was collected as SlitCSP sera^24^. The serum titer was showed to have an enzyme linked immunosorbent assay (ELISA) end point of 1:12, 000 using the method of indirect ELISA^25^. Western-blotting analysis was modified according to the methods previously described^26^. Samples were electrophoresed on 12% SDS polyacrylamide mini-gels and transferred to PVDF membranes using Tris–glycine transfer buffer on a mini-Trans-Blot electrophoretic transfer tank (Bio-RAD, USA). Blots were blocked in TBS (100 mM Tris–HCl, pH 7.5, 0.9% NaCl) containing 5% nonfat powdered milk and 0.1% Tween-20 for 1 h. The immunoreactivity was tested with the anti-SlitCSP serum (diluted 1: 5000), and incubated with the filter overnight at 4 °C. Blots were washed with TBST three times. An IgG anti-rabbit antibody conjugated with HRP was used as a secondary antibody (Tiangen, China) and finally visualized by ECL (enhanced chemiluminescence).

### Fluorescence competitive Binding Assays

The fluorescence spectra were recorded on an F-4500 FL Fluorescence Spectrophotometer (HITACHI) in a 1 cm light path quartz cuvette at 23 °C. The slit width used for excitation and emission was 5 nm. The compounds used to investigate the binding abilities of SlitCSPs were purchased from Sigma-Aldrich with the highest purity and stored as specified instruction by the manufacturer. The selected chemicals were listed in Table 2, which are the typical volatiles of Chinese cabbage (*Brassica camperstris ssp.pekinens*) and tobacco (*Nicotiana tabacum L.*) based on previous investigations. The fluorescent probe N-phenyl-1-naphthylamine (1 − NPN) and all ligands used in competition experiments were dissolved in HPLC purity grade methanol. To measure the affinities of 1 − NPN to three purified SlitCSPs protein, the fluorescence of 2 μM 1 − NPN in 50 mM Tris-HCl was excited at 337 nm and emission spectra were recorded between 350 nm and 480 nm. And then, 2 μM of protein was added and titrated with aliquots of 1 mM 1 − NPN to final concentrations of 2 to 20 μM. The affinities of the chemicals were measured by competitive binding assays in presence of three candidate SlitCSPs protein at 2 μM and 1 − NPN at 4 μM by adding ligands from 0 to 20 μM. All values reported were obtained from three independent measurements. The corresponding to the maximum fluorescence emission was plotted against the ligand concentrations for the determination of the binding constants. The curves were linearized by Scatchard plots. The dissociation constants of the competitors were calculated using the corresponding IC_50_ values according to the equation: KD = [IC_50_]/(1 + [1 − NPN]/K_1−NPN_), where [1 − NPN] is the free concentration of 1 − NPN and K_1−NPN_ is the dissociation constant of the complex protein/1 − NPN^27^.

**Table 2:** Fluorescence competitive binding affinities of selected components to recombinant three SlitCSPs.

IUPAC Name	CAS No.	Resource	IC_50_			*K*d		
			SlitCSP11	SlitCSP3	SlitCSP8	SlitCSP11	SlitCSP3	SlitCSP8
**Aliphatic alcohols**								
cis-3-Hexen-1-ol	928-96-1	Green leaf volatile^a^	u.d	u.d	5.68	u.d	u.d	8.07
**Aliphatic aldehydes**								
Hexanal	66-25-1	Cabbage^b^	u.d	u.d	u.d	u.d	u.d	u.d
**Aliphatic ketones**								
6,10-Dimethyl-5,9-undecadien-2-one	689-67-8	Cabbage^c^	7.16	5.41	7.09	12.17	8.65	10.70
**Aliphatic acid**								
trans-2-Hexenoic acid	13419-69-7	Tobacco^d^	u.d	19.33	5.59	u.d	9.74	7.94
Hexadecanoic acid	57-10-3	Tobacco^e^	3.42	u.d	u.d	5.82	u.d	u.d
**Aliphatic esters**								
Butyl isothiocyanate	592-82-5	Cabbage^f^	12.12	u.d	u.d	20.59	u.d	u.d
Isothiocyanicacid	556-61-6	Cabbage^g^	11.92	u.d	u.d	20.26	u.d	u.d
**Aromatic alcohols**								
Benzyl alcohol	100-51-6	Tobacco^i^	5.28	u.d	7.89	8.97	u.d	11.20
**alkene**								
Styrene	100-42-5	Cabbage^j^	13.78	u.d	u.d	23.40	u.d	u.d
Menthol	2216-51-5	Tobacco^k^	4.11	6.19	ud	6.99	9.90	u.d
Phenol	108-95-2	Cabbage^l^	5.90	5.05	u.d	10.03	8.07	u.d
**Aromatic esters**								
Phenethyl isothiocyanate	2257-09-2	Cabbage^m^	5.32	5.67	4.34	9.04	9.07	6.17
**Aromatic ketones**								
5-(Hydroxymethyl)furfural	67-47-0	Cabbage^n^	u.d	4.49	u.d	u.d	7.18	u.d
Furan-2-carboxaldehyde	98-01-1	Tobacco°	14.08	u.d	u.d	23.93	u.d	u.d
Benzaldehyde	100-52-7	Tobacco^p^	9.55	4.85	5.26	16.22	7.75	7.47
β-Ionone	14901-07-6	Cabbage^q^	2.90	7.16	3.57	4.93	11.44	5.07
**Heterocyclic compound**								
2-Ethylfuran	3208-16-0	Cabbage^r^	u.d	u.d	u.d	u.d	u.d	u.d
Pyridine	110-86-1	Tobacco^s^ Cabbage^t^	6.79	u.d	u.d	11.54	u.d	u.d
Isonicotinamide	1453-82-3	Tobacco^u^	15.49	6.27	6.25	26.32	10.02	8.88
**Others**								
Benzonitrile	100-47-0	Cabbage^v^	u.d	u.d	u.d	u.d	u.d	u.d
Decanenitrile	1975-78-6	Cabbage^w^	u.d	u.d	u.d	u.d	u.d	u.d

Solution of protein was at 2 μM, and the added concentration of 1 − NPN was in line with the dissociation constants of SlitCSPs/1 − NPN complex calculated. Then the mixed solution was titrated with 1 mM solution of each ligand in methanol to final concentrations of 0 to 20 μM. *K*_D_ = dissociation constant of the competitors; IC_50_ = competitor’s concentration halving the initial fluorescence. Dissociation constants of ligands whose IC_50_ exceeded 50 μM are represented as “kd”. ^a b c d e f g h i j k l m n^References: ^a^Ruther, J. *et al.*, *Journal of chemical ecology* 2005, *31* (9), 2217–2222. ^b^Song T. Y. *et al.*, *Food science*, 2010. 31(8): 185–188. ^c^Zhao D. Y. *et al.*, *Food science and technology*., 2007, 40(3): 439–447. ^d^Guo L. *et al.*, *J of instrumental analysi*s 2008–07. ^e^Sun S. H. J of chromatography A. 2008 1179(2) 89–95. ^f^Bettery R. G. *et al.*, J. *agri food chem* 1976. 24(4) 829–823. ^g^Wu C. Y. *Shandong agricultural university*. 2008. ^i^Ribnicky D. M. *et al.*, *Plant physology* 1998 118(2) 565–572. ^j^Truchon G. *et al.*, *Journal of occupational health*. 1998 40(4): 350–355. ^k^Gandhi K. K. 2009 63(3) 360–367. ^l^Hendrich S. *et al.*, *Food and chemical toxicology*., 1983 21(4) 479–486. ^m^Wu C. Y. *et al. Food science*., 2009 (4). ^n^Kim D. O. *et al.*, *Journal of food science* 2006 69(9) 395–400. ^o^Wu L. J., *Anal Methods*, 2013 5:1259–1263. ^p^Clark T. J., *Journal of agricultural and food chemistry* 1997 45(3) 844–849. ^q^Lonchamp J. *et al.*, *Food research international*. 2009 42(8) 1077–1086. ^r^Song T. Y. *et al.*, *Food science*, 2010. 31(8): 185–188. ^s^Stepanov I. *et al.*, *Cancer epidemiology biomarkers & prevention* 2005 14:885. ^t^Takasugi M. *et al.*, *Bulletin of the chemical society of Japan*. 1988 61(1) 285–289. ^u^Taguchi H., *Bioscience*, *biotechnology and biochemistry*. 1997 61(4). ^v^Kobayashi M. *et al.*, FEMS microbiology letters. 1994. 1:217–223. ^w^Machiels D. *et al.*, Talanta 2003, 60(4): 755–764.

### Statistical analysis

All the results from experimental replicates ere expressed as the mean (±S.E.M.) and analyzed by one-way analysis of variance (ANOVA) and *t*-test using SPSS 17.0 for Windows software (SPSS Inc, Chicago).

## Result

### Identification Midgut Expressed Chemosensory Proteins in *S. litura*

**Figure 1:**
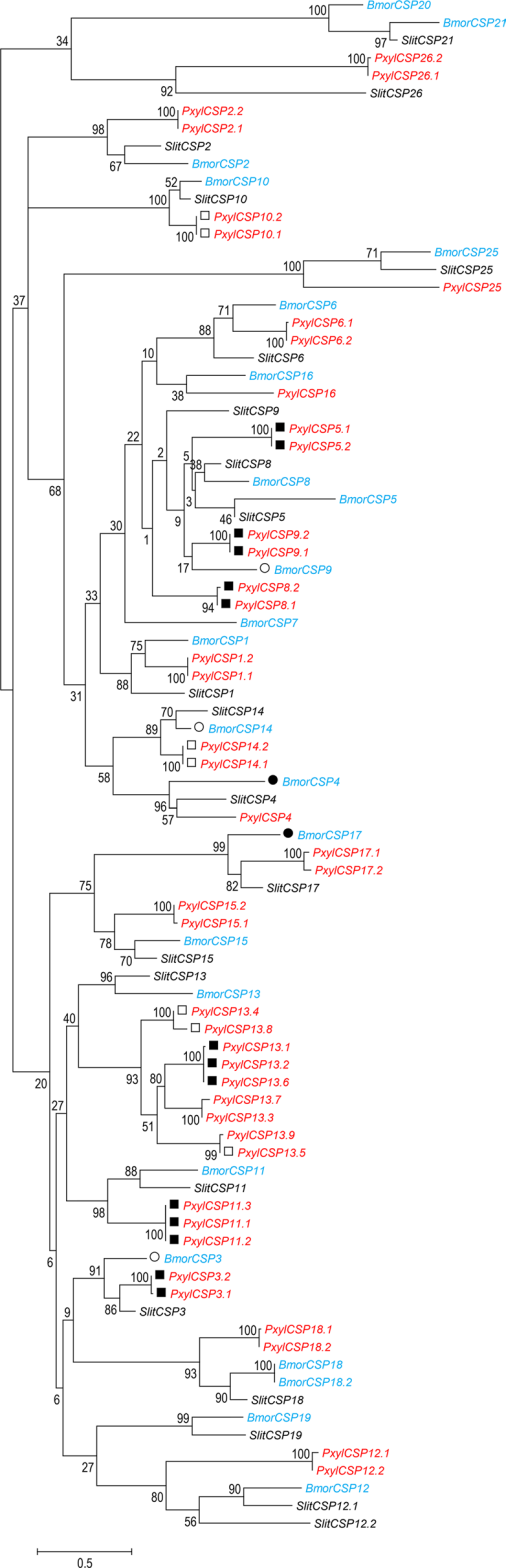
Phylogenetic analysis of chemosensory protein in three Lepidoptera insects. The phylogenetic tree was constructed in MEGA 6.0 using neighbour-joining method. Bootstrap values >50% (1000 replicates) are indicated at the nodes. ● The results showed the RPKM values of BmorCSP4, BmorCSP17 from two or more transcriptome data could reach to top 25%. ○ The RPKM values of BmorCSP3, BmorCSP9, BmorCSP14 from at least one transcriptome dataset were above average value. ■ The RPKM values of PxylCSP4, PxylCSP5, PxylCSP7, PxylCSP8, PxylCSP13, PxylCSP22, PxylCSP23, PxylCSP24, PxylCSP26, PxylCSP27, PxylCSP32, PxylCSP41, PxylCSP42, PxylCSP43 from two or more transcriptome dataset could reach to top 25%, which indicated their high expression were reliable. □ The RPKM values of PxylCSP10 and PxylCSP29 from at least one transcriptome dataset were above average value.

**Figure 2:**
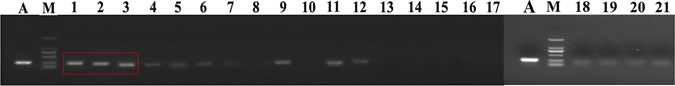
Expression levels of identified SlitCSPs in midguts by RT-PCR. Detection of identified SlitCSPs in midguts by RT-PCR. A: actin gene of *S. litura.* M: DNA maker. 1, SlitCSP11, SlitCSP3 3: SlitCSP8 4: SlitCSP5 5: SlitCSP12.1; SlitCSP12.2; 7, SlitCSP6; 8, SlitCSP18; 9: SlitCSP19; 10, SlitCSP1; 11, SlitCSP14; 12: SlitCSP15; 13: SlitCSP9; 14: SlitCSP17; 15, SlitCSP13; 16: SlitCSP18; 17: SlitCSP2; 18: SlitCSP26; 19: SlitCSP10; 20: SlitCSP25; 21: SlitCSP4. The red box indicated three candidate CSPs were selected to subsequent study.

By analyzing the *de novo* transcriptome assembly of *S. litura* we reported previously^18^, we identified 21 non-redundant CSP coding transcripts (hereafter referred to as SlitCSP genes) (Table 1S, Fig. 1) including 14 SlitCSPs characterized in another transcriptome study^28^ and seven newly discovered ones. We then performed RT-PCR to determine the midgut expression levels of these CSPs. As shown in Fig. 2, 14 of the 21 SlitCSPs had detectable expression in midgut. Among them, SlitCSP11, SlitCSP3 and SlitCSP8 could express highly in midgut. Therefore, these CSPs (SlitCSP11, SlitCSP3 and SlitCSP8) were selected for further studies.

To compare the midgut expressed CSPs in *S. litura* with that in other closely related insects, we also identified 23 *CSP* genes in (BmorCSPs) and 43 CSP genes in *P. xylostella* (PxylCSPs) from previous reported genome assemblies compared with previous reports^29, 30^, these include one new BmorCSP and 11 new PxylCSPs. Phylogenetic analysis of all 87 *CSP*s from the three insect revealed 17 well-supported clades (bootstrap support ≥70%) displaying clear orthologous relationships between CSPs from different species (Fig. 1). Almost all these clades consist of a single effective gene (or group of very recent duplicates in the case of *P. xylostella*) from each species. Interestingly, the *B. mori* and *P. xylostella* orthologs of SlitCSP3, SlitCSP8, and SlitCSP11 are all highly expressed in midgut (Fig. 1).

### Expression pattern of three SlitCSPs

**Figure 3:**
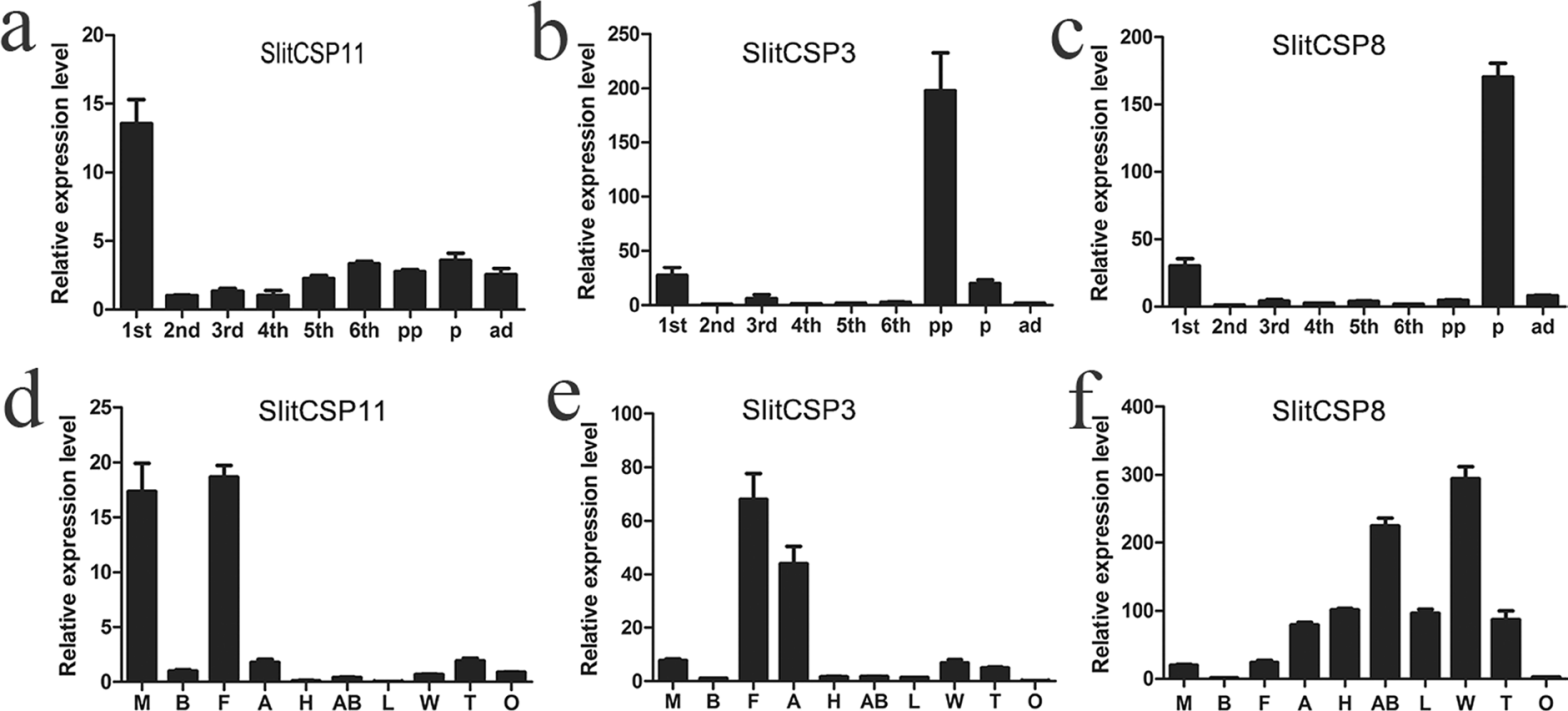
Quantitative real-time RT-PCR analysis the expression pattern of three CSPs in *S. litura*. (**a**–**c**) Different developmental stages: 1^st^: 1st instar larva; 2^nd^: 2^nd^ instar larva; 3^rd^: 3^rd^ instar larva; 4^th^: instar larva; 5^th^: 5^th^ instar larva; 6^th^: 6^th^ instar larva; pp: pre-pupae, p: pupae, ad: adult. (**d**–**f**) Different adult tissues: M: midguts; B: body wall; F: fat body; A: antenna; H: head (without antenna); AB: abdomen; L: leg; W: wing; T: testis; O: ovary. (**a**,**d**,**b**,**e**,**c** and **f**) represent the SlitCSP11, SlitCSP3 and SlitCSP8 respectively.

Three SlitCSPs with highest midgut expression levels (SlitCSP11, SlitCSP3 and SlitCSP8) were selected for in-depth analysis of their expression patterns. For *S. litura*, early instar larvae were fed on the lower part of leaf layers. For 2^nd^ and 3^rd^ instar larvae of *S. litura*, they started to proliferation. And from 4^th^ to 6^th^ instar larvae, the moths start to enter one period of gluttony, that is, the insect could eat many kinds of plants without special selectivity^31^. As its relatively stable expression level of 2^nd^ instar larvae, RT-qPCR was used to investigate the expression levels of these three SlitCSPs in various developmental stages and tissues by using the 2^nd^ instar larvae sample as the calibrator^32^. Although SlitCSPs were found to be expressed at multiple stages, their expression levels varied greatly from each other. For SlitCSP11, the highest expression was observed at the 1^st^ instar larva, reaching to 13.56-fold higher compared with the 2^nd^ instar larvae. The highest expression level of SlitCSP3 was observed in pre-pupae, which was 195.87-fold higher than that of the 2^nd^ instar larvae. Other high expression level of SlitCSP3 was observed in 1^st^ instar larvae and pupae, reached to 26.95-fold and 19.61-fold higher compared with 2^nd^ instar larvae. Likewise, high expression of SlitCSP8 was also observed in 1^st^ instar larvae, which was 30.28-fold higher than 2^nd^ instar larvae. However, the highest expression level of SlitCSP8 was observed in pupae, which is 170.40-fold higher than 2^nd^ instar larvae (Fig. 3a).

The expression levels of three candidate SlitCSPs in various tissues were also examined, including cuticle, midguts, fatbody, antennae, heads (without antennae), wings, legs, abdomens, testis and ovary (Fig. 3b). Besides high expression in the chemosensory organs, these three SlitCSPs were also expressed in non-chemosensory organs, while the highest expression was observed in cuticle, fatbody and midguts. The SlitCSP3 was expressed in both chemosensory and non-chemosensory organs, namely, fatbody and antennae. However, unlike SlitCSP11 and SlitCSP3, the SlitCSP8 was expressed in all tested tissues except ovary and cuticle.

### CSPs expression level analysis after different treatments

**Figure 4:**
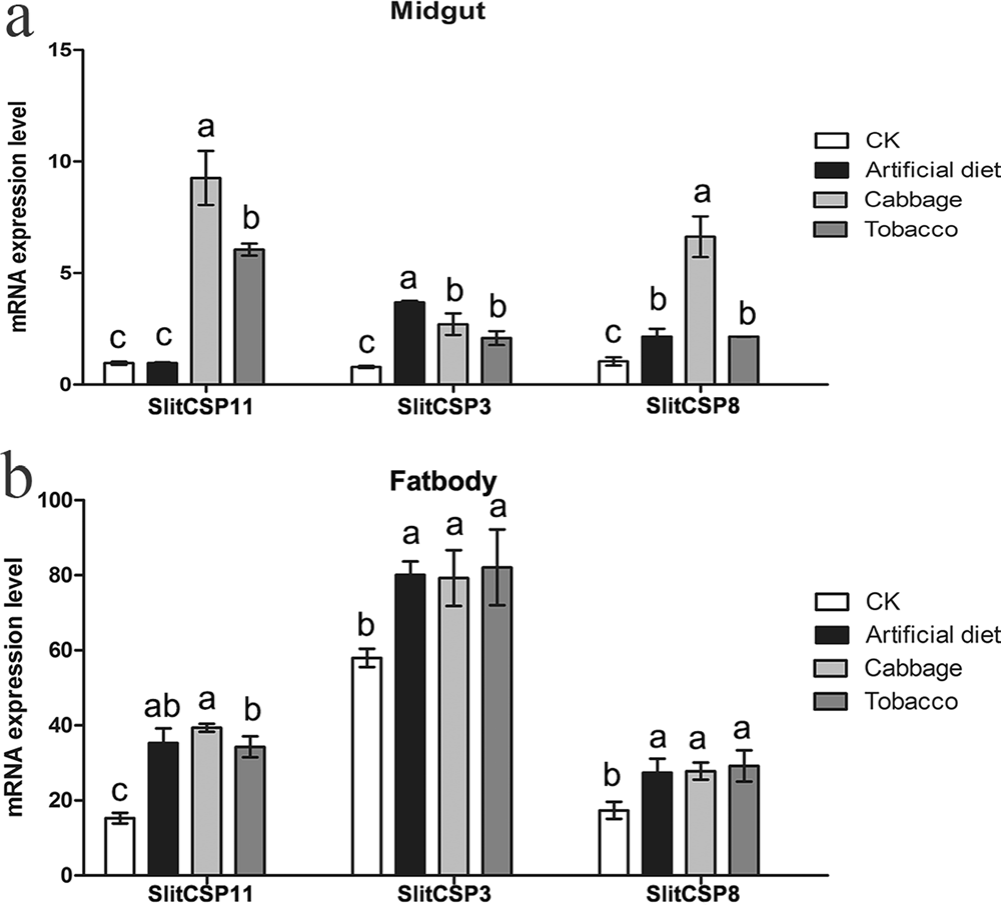
Relative expression of SlitCSPs after different treatments in midgut and fatbody. (**a**) midgut; (**b**) fatbody. CK was negative control, which fed on nothing. 1, SlitCSP11; 2, SlitCSP3; 3, SlitCSP8. All the data represent the mean values ± S.E.M. of replicates. Different letters indicated significant differences of expression levels of candidate CSPs between the treated by treatments and CK, as determined using a *t*-test (*p* < 0.05).

Three SlitCSPs were examined for expression variations in the midgut of fourth instar larvae upon feeding with different diets. After treatment, the survival rate for the treatment group and control group are 100%. The results showed that the expression of SlitCSP11 in midgut was up-regulated by 2.15-fold, 6.63-fold and 2.16-fold after the moths were fed with artificial diet, cabbage and tobacco, respectively (Fig. 4). When compared with negative control, the expression of SlitCSP3 in midgut was up-regulated by 3.68-fold, 2.71-fold and 2.09-fold after the treatment of artificial diet, cabbage and tobacco, respectively. The expression of SlitCSP8 in midgut was up-regulated by 9.26-fold and 6.05-fold after fed with cabbage and tobacco respectively. As control, the expression levels of CSPs in fat body increased compared with the starvation group, however, significant differences could not be observed among different treatments (Fig. 4).

### Fluorescence Binding Assays

The result showed that three SlitCSPs recombinant protein were successfully induced and expressed (Supplementary Fig. 1a–c). These SlitCSPs were resolved as a single band with molecular weight of around 33 kDa by western blot (Supplementary Fig. 1d), and then the proteins were purified successfully (Supplementary Fig. 1e). After subjected to the Bovine Enterokinase, the result of SDS-PAGE showed the molecular weight of the recombinant protein SlitCSP11, SlitCSP3 and SlitCSP8 was 12.1, 13.9, and 14.9 KDa, respectively, after removed the His-tag (18 kD) successfully (Supplementary Fig. 1f). Three proteins could be used for further investigation.

**Figure 5:**
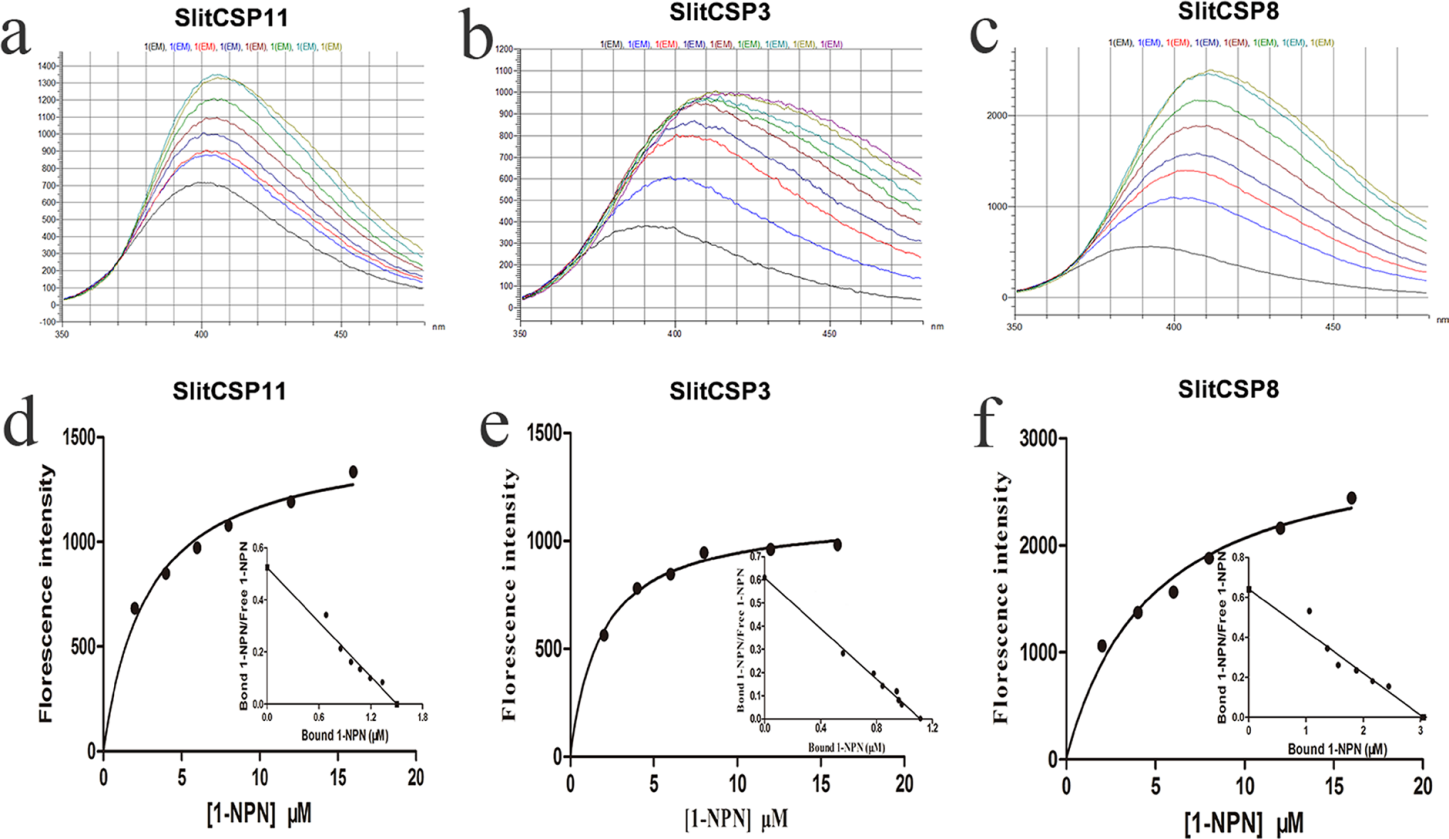
Ligand-binding assays of the three SlitCSPs. (**a**,**b**,**c**) Binding curve for different concentration of 1 − NPN to SlitCSP11, SlitCSP3 and SlitCSP8. (**d**,**e**,**f**) Scatchard plot of these three CSPs. The binding curve of 1 − NPN and relative Scatchard plot analysis (insert). To measure the affinity of 1 − NPN to three SlitCSPs, the fluorescence of 2 μM 1 − NPN in 50 mM Tris-HCl was excited at 337 nm and emission spectra were recorded between 350 nm and 480 nm. Then, 2 μM of protein was added and titrated with aliquots of 1 mM 1 − NPN to final concentrations of 2 to 20 μM. The experiment was replicated for at least three times, and the data were analyzed using Prism software and indicated the presence of a single binding site. The solution was excited at 337 nm.

**Figure 6:**
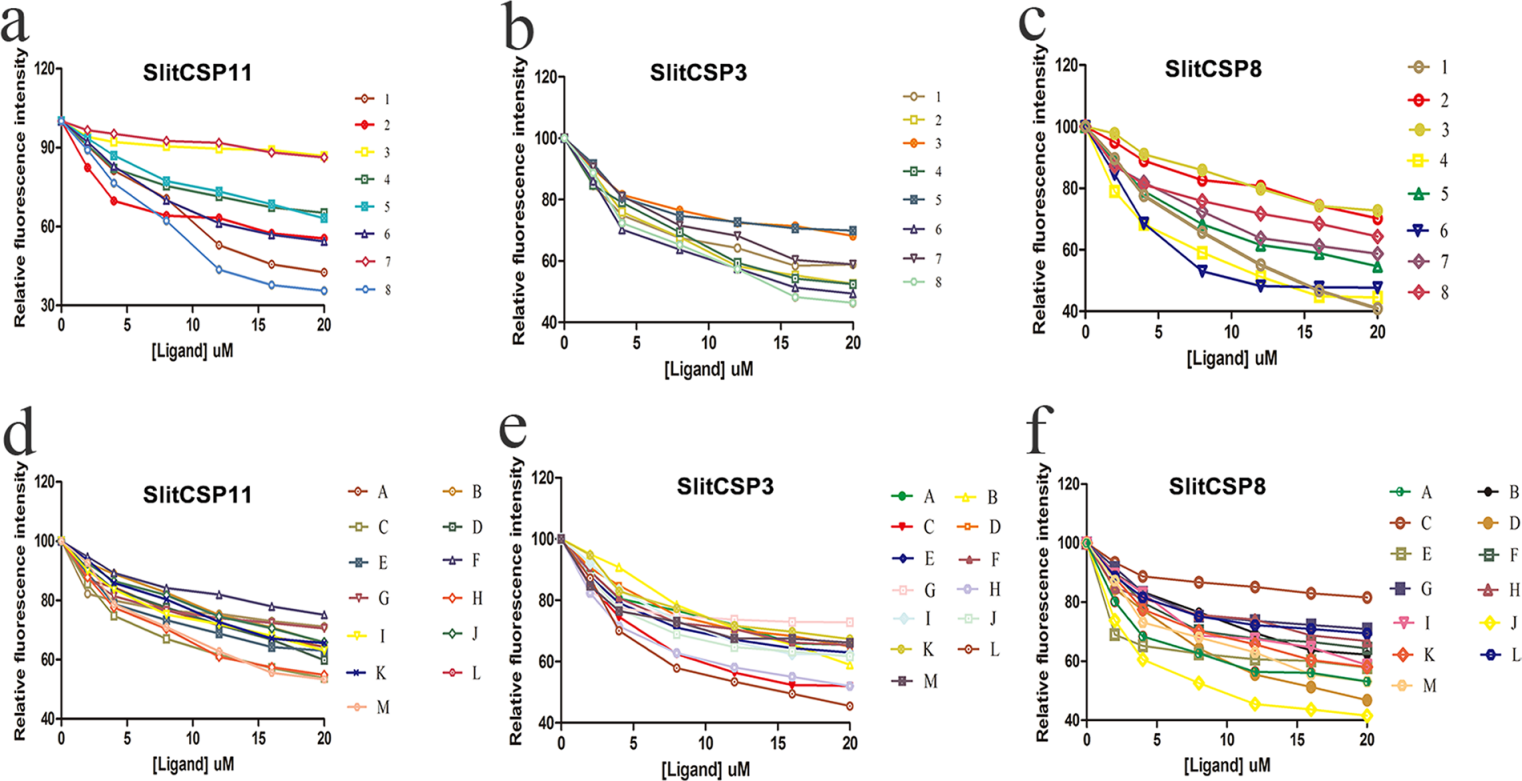
Competitive binding activities of the selected ligands with three candidate SlitCSPs. (**a**) SlitCSP11; (**b**) SlitCSP3; (**c**) SlitCSP8. 1: Competitive binding activities of the candidate protein with typical odor chemicals from tobacco. 1: β-Ionone; 2: Hexadecanoic acid; 3: Menthol; 4: Isonicotinamide; 5: Benzyl alcohol; 6: Benzaldehyde; 7: Pyridine; 8: Furan-2-carboxaldehyde; 2: Competitive binding activities of the candidate protein with typical odor chemicals from cabbage. A: cis-3-Hexen-1-ol; B: 2-Ethylfuran; C: Phenol; D: Phenethyl isothiocyanate; E: Styrene; F: Benzonitrile; G: Decanenitrile; H: Hexanal; I: 5-(Hydroxymethyl)furfural; J: trans-2-Hexenoic acid; K: Butyl isothiocyanate; L: Isothiocyanicacid; M: 6,10-Dimethyl-5,9-undecadien-2-one.

The protein was expressed with good yield (20.5 μg/μl, 12.3 μg/μl, 22.7 μg/μl). By titrating the SlitCSPs with increasing concentration of 1 − NPN, a saturation (Fig. 5a) and linear Scatchard plot were observed (Fig. 5a), indicating a single binding site and no allosteric effect. Three SlitCSPs could bind to the probe with dissociation constants of 2.860 μM, 3.337 μM and 4.756 μM (Fig. 5b). By using 1 − NPN as the fluorescent reporter, the affinities of SlitCSPs to a series of compounds were measured in competitive binding assays (Fig. 6). The IC_50_ values (the concentration of the ligand that yielded 50% of the initial fluorescence value) and calculated binding constants were reported in Table 2. From the results, the SlitCSP8 showed better binding activities with the typical odors from both cabbage and tobacco when compared with SlitCSP11 and SlitCSP3. Many chemicals could complete the 1 − NPN from the binding site of SlitCSP8 at low concentrations, namely, cis-3-Hexen-1-ol, benzyl alcohol and isonicotinamide. Most of the selected ligands could bind to the SlitCSP8, except for Hexanal, 2-Ethylfuran, Benzonitrile and Decanonitrile, which showed poor affinities with all the tested protein. For SlitCSP11, it showed high affinities with the five compounds of tobacco and three compounds of cabbage. SlitCSP3 had good binding activities with four ligands of tobacco and four ligands of cabbage.

## Discussions

Midgut, as a dynamic tissue, was suggested to play a vital role in metabolism, digestion and detoxification^33, 34^. In Lepidoptera, previous studies have focused on the role of proteases, lipases and carbohydrases in digestion, carboxylesterases, glutathione-S-transferases and cytochrome P450s in midgut^35–37^. Other than those, our study focused on physiological roles of chemosensory-related protein in midgut. In the present study, we identified 21 CSPs from the transcriptome assembly of *S. litura*, 23 CSPs from the genome assembly of *B. mori* and 43 CSPs from the genome assembly of *P. xylostella* (Fig. 1). Midgut expression levels of CSPs in *S. litura* were examined by RT-PCR. Our results showed that not all the identified CSPs could be detected to be expressed in midgut (Fig. 2). The same phenomenon was observed in *Bactrocera dorsalis*, among the identified four CSPs in *B. dorsalis*, only two of them could be detected in abdomen^38^.

Three candidate CSPs (SlitCSP11, SlitCSP3, SlitCSP8), which showed highest expression levels in midguts, were selected to further investigation. These three CSPs expressed highly in early pupae (Fig. 3), which implied their function in chemoreception in this period^39, 40^. Consistent with our results here, a previous RNA sequencing (RNAseq) study in *S. litura* revealed that the expression levels of many SlitCSPs could be enhanced in whole insect bodies, including thorax, wings, labial palps, tarsi, proboscis, pheromone glands and ejaculatory ducts^38, 41, 42^. One interpretation of those data is that SlitCSPs are functional in nonhead tissues where they are used in noncanonical chemosensory roles. In fact, these proteins have been shown to be involved in development^43, 44^ and immune protection^44^. Other similarly fancy experiments in moths have suggested a role of CSP as wetting agent to reduce the surface tension of aqueous sugar solutions and thereby reduce the pressure involved in sucking nectar^45^.

As previous reports suggested that the repertoires of chemosensory related proteins, especially CSPs, could be under the selection pressure that is influenced by the ecological status of different insect species^46^. Comparative gene expression studies enable the identification of biological functions involved in the adaptation of organisms to their surrounding environments^15^. By proteomics approach, Celorio-Mancera *et al.* demonstrated the expression of CSP1 changed in response to the caterpillar diet in the mandibular glands. In the meanwhile, the CSP2 abundances also changed in both labial and mandibular glands after changed to different diet resources^16^. Some CSPs might also act in a sort of immune protection in gut against insecticides, as their gene expressions have been reported to be upregulated in the gut of some insect species by such insecticides^47, 48^. Fourteen CSP genes in the silkworm moth were significantly up-regulated in various non-chemosensory tissues in response to avermectins, which suggested the roles of such protein in xenobiotic degradation and insect defense in the whole body^48^. In this study, the expression levels of three SlitCSPs in midgut were regulated when switched to different feeding resources (Fig. 4), which suggested those CSPs could be functional in midgut. This may be the case when these proteins act as sequestering agents for noxious compounds or as nutrient solubilisers, but it is also likely that they might play the role of carriers for specific hormones^49^. The function of CSPs in regulating nutrient signals is also supported by the fact that the identification of abundant CSPs in the proboscis of Lepidoptera, which was suggested to be involved in helping solubilizing important hydrophobic nutrients^49^. In this study, the changes in expression levels of CSPs in midguts in response to different diets provided us with initial evidence, which could support our hypothesis that CSPs may play roles in midguts and may eventually control insect behaviors by influencing nutrient utilization or inhibiting appetite. The detailed mechanism of CSPs in midgut needs further studies.

All these functions, although unrelated to chemical communication, may still be linked to the binding capacities of CSPs for all sorts of hydrophobic chemicals. Generally consistent with bioassay, in the fluorescence binding assays in this study, SlitCSP6 also showed good binding activities with typical volatile signals from cabbage. The SlitCSP8 could bind well with typical chemicals from tobacco, and when the *S. litura* was fed by tobacco, the abundance of SlitCSP8 in midgut was up-regulated. However, when fed with Cabbage, the expression level of SlitCSP8 did not show significant change. This may be due to the reason that the SlitCSP8 could not bind well with typical odors from cabbage in competitive binding assays (Table 2). Different host plants may cause changes in expression levels of CSPs, due to their recognition abilities and binding preferences to different chemical signals from different food resources. The internal hydrophobic binding cavity composed of α-helices, which could bind the relevant chemicals, enabled CSPs execute such function^50^.

Overall, 21 CSPs from *S. litura* were identified in this study. Moreover, the expression variations of CSPs in midguts responding to different diet treatments and the binding affinities between CSPs and typical odors from host plants were investigated. The results of this study could preclude or reinforce that the changes of CSP expression levels in midgut may act in concert to modulate host specialization to adapt different ecosystem.

## Electronic supplementary material

Supplementary information
