# Supplementary material for: Differential expression of chemosensory-protein genes in midguts in response to diet of Spodoptera litura
Source: Sci Rep. 2017 Mar 22;7:296. doi: 10.1038/s41598-017-00403-5 (PMC5428418; doi:10.1038/s41598-017-00403-5)
Supplement: Supplementary file 1 — Supplementary information [file 41598_2017_403_MOESM1_ESM.pdf]

Differential expression of chemosensory-protein genes in midguts in response to diet of *Spodoptera litura*

Xin Yi[#1], Jiangwei Qi[#1], Xiaofan Zhou[#2], MeiYing Hu[1], GuoHua Zhong[*1]

1 Laboratory of Insect Toxicology, Key Laboratory of Pesticide and Chemical Biology, Ministry of Education, South China Agricultural University, Guangzhou, People's Republic of China

2 Guangdong Province Key Laboratory of Microbial Signals and Disease Control, Integrative Microbiology Research Centre, College of Agriculture, South China Agricultural University, Guangzhou, China

---

[#] All authors contributed equally to this work.

[*] Correspondence to: Prof GuoHua Zhong. Key Laboratory of Pesticide and Chemical Biology, Ministry of Education, PR China, College of Natural Resources and Environment, South China Agricultural University, Guangzhou 510642, China. E-mail: guohuazhong@scau.edu.cn. Tel: +86-20-85280308; Fax: +86-20-85280292

Supplementary Fig. 1 Expression and purification of three candidate SlitCSPs

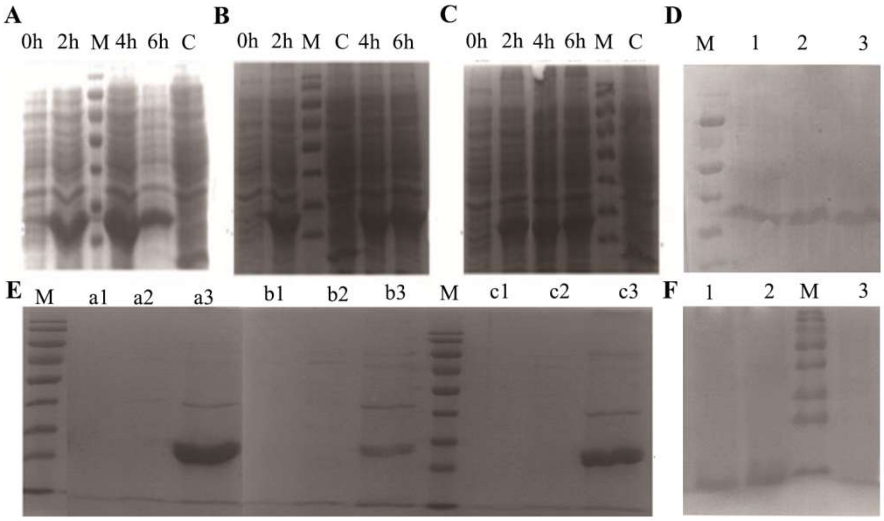

A, 12% SDS-PAGE of the expressed fusion protein of SlitCSP6; 0h: 0h IPTG inducing of fusion protein; 2h: 2h-inducing of fusion protein; M: molecular weight marker of 10, 15, 25, 35, 40, 55, 70, 100, 130 and 170 KDa. C: empty vector; B, 12% SDS-PAGE of the expressed fusion protein of SlitCSP1; 0h: 0h IPTG inducing of fusion protein; 2h: 2h-inducing of fusion protein; M: molecular weight marker of 10, 15, 25, 35, 40, 55, 70, 100, 130 and 170 KDa. C: empty vector; C, 12% SDS-PAGE of the expressed fusion protein of SlitCSP2; 0h: 0h IPTG inducing of fusion protein; 2h: 2h-inducing of fusion protein; M: molecular weight marker of 10, 15, 25, 35, 40, 55, 70, 100, 130 and 170 KDa. C: empty vector; D, Western blot results of purification of the protein. M: molecular weight marker of 10, 15, 25, 35, 40, 55, 70, 100, 130 and 170 KDa; 1: SlitCSP11; 2: SlitCSP3; 3: SlitCSP8. E, Purification was accomplished by HisTrap affinity columns. M: molecular weight marker of 10, 15, 25, 35, 40, 55, 70, 100, 130 and 170 KDa; a: SlitCSP11; b: SlitCSP3; c: SlitCSP8; 1: the protein sample was washed by buffer B; 2: the protein sample was washed by buffer C; 3: the protein sample was washed by buffer E. F: 12% SDS-PAGE analysis after the protein was subjected to Bovine Enterokinase overnight to remove the His-tag. M: molecular weight marker of 10, 15, 25, 35, 40, 55, 70, 100, 130 and 170 KDa; 1: SlitCSP11; 2: SlitCSP3; 3: SlitCSP8.

Table 1S Chemosensory protein identified in fasta format
> comp22025_c0 (SlitCSP21)
TCGTTGGTCGGACGTAACCTAAATATTCGGCTCCGTTTCTACTACAACTTCAGTGATAA
A
ATATTGAAATTAAATCCGTGCAAAGTGGGCGTTTCTATAATGCAGATCATCGTAGTTTTT
GTGGTGGCGTGTGTGGGTTTGGTAGCCGGCTTGCATGTGCAGGCTGGGCCACAAATGA
CA
GACGCACAATTAGAACAGACTTTAGCCGATAAGAGCACGATGCAGCGACACATTAAAT
GC
GCACTCGGTGAAGGACCTTGCGATCCTGTTGGGAGACGATTACGAACTTTAGCCCCGT
TA
GTACTACGAGGGGCCTGTCCACAATGTTCTATGCAGGAGACGCGCCAGATTCGACGCA
CC
CTTGCCTTCGTACAACGCAACTACCCATGGGAGTGGGCGAAAATTGTTCGCCAATACG
GT
TAA
> comp19189_c0 (SlitCSP26)
AGGTGTTGTTCTTCAGTGTTATTCATTGTATCAGAGATTAACCAAAGCTTGAAGATGAA
G
GTACTAGTAGCGTTGTCCATATTCGTCGTACTAGCAGCAGCAGTACCAATTACAAAGGA
T
GAGTTCGCTACACTTGAGGCATTCGACTATGACGCCCTATTCGCTGATGAAGAGAACA
GA
AAAATGGTCTTCGATTGCTTGCTAGATAAAGGAGATTGTGGTCCTTACAAACAAATCGT
T
GAGCTGTCAATGAAAATAATACTTAGCAACTGTGCAGAATGTTCTCCATCCCAGAAGGC
G
AAATACGATCACGTACTAAAAGTATTGAAGGAAGACTATGCGTCCTTCTTCAATGAACT
C
ATGCAGAAAGCTGCTTCCAAAAAGGAAAAACATTAA
> comp18805_c0 (SlitCSP2)
ATAAATATAGGACAGGTCGCCGAGTACAGCACTCAGCTGTTAGACAGTGATCGAGATTC
A
CCTATCAGTGTAAGGAGGCCAGTAATCATGAAGGTAGCGCTACTCACACTCTGCTTCGC
T
CTTGGAGTCCTCGCACAGGATATGTACGAGAATGCCAACGACAACTTTGACATCTCTG
AG
GTTCTGGGCAATGAAAGGCTCCTCAACTCCTATGCTAAGTGCCTACTCAACAAGGGAC
CT
TGCACTCCAGAAGTAAAGCAAGTTAAAGATAAATTGCCGGAAGCTCTGGAAACTCGGT
GT
GCAAAATGTACGGATAAACAAAAGCAAATGGGCAAGACTTTGGCTCAAGAAGTGAAG
AAA
AATCATCCCGACATCTGGAAGCAACTGGTGGCAATGTACGACCCTGAAGGCAAATACC

AA
CAAGCGTGGAAGGACTTCCTCCAAGAATAA
> comp21913_c0 (SlitCSP10)
GTTTTTAGTTTAACTAGAAATAATAGATTAAAAATGCGTGCAGTGTTGTTACTTTGTGCT
CTAGTGCATTTAGTGGTGGGACAAGATGTTAATGACATGGTAAACATGCCTAAATACGA
T
CAGAGATACGATTATTTGGACGTCGATGCAATTTTCACGAACAAGAGACTCGTTAGGA
AC
TATGTGGATTGTTTGATTAATGCTGTTCGGTGCACGCCTGAAGGAAAAGCGCTGAAAC
GA
ATACTCCCAGAAGCGTTGAGGACGAAATGTGTCCGCTGCACAGAGCGACAGAAGAGA
ACA
GCAGTGAAGGTGATCAAGCGGCTGAAGAACGAATATCCTGACGAATGGTCCAAGCTTG
CT
TCCCGGTGGGACCCCACTGGGGACTTCACCAGATACTTCGAAGAATTCCTCGCTAAAG
AA
CACTATAACACCATCCCAGGATCAGGCAGTGCGCTCCCTACGTCATCACCGTTAGCACC
A
CCACGTGTACCACAATCACCACCAGCTACCAACCCGACTCCAGGCCCCACGGAGCCTA
CC
CCACCGAGGCCTATTGTACTGAACAGGTTTGGTGACGACGGCGAGCTGATGATGGGCA
GT
CCATCGTCAGCGGGTATTACTCCGAGACCGATGACTCAGGCCACTACGAGACCCAGCA
CT
ACCACGAGGCCGCCTAGTACTAGGCCTGTACCACCCAGACCGACAATGATGACATGGG
CT
GGTGCAGCGTCCAACACGCAGGCGACGCGTTTCCCATTGCGGCCTGTATCAGAAATAT
CA
CCACCATACTCCACGGCCATAACCCTCATCGACCAGATCGGATACAAAATAATAAAGAC
A
ACAGAACTTGTCACCGATTTACTCAGGAACACCGTTAGAGCTGTCGTTGGTCGGTAG
> comp13897_c0 (SlitCSP25)
GAGCTATCAATGGTCTCAGTCATAATGTGGGCTCAAGCGATTCCGTACTTGATTGTTGTA
CATGTGGTGATGGGAGATCAGGATATGCCTGGGTTCGTGAGAGAAATGAGCGATGGTG
TA
GAGAGTAGAGGCTTCAAGATCATTTACGGCGATGAAGACATGACCATTATCAACCAAG
TG
GTCAGTGAAGCCGAAAAAGGCAGTGCTTTTAAGAAGAGAGCACATTTAAATGAGGCTA
TT
AAGCCCTTACCAGCGCAAGATGTCAAGTGCTTGATGTCTGTGGATCGGTACTGTAGCA
AG
GAGATGGGGCTGATGAAAAGTGTATTGATACAAGCTGTAAAAGATGACTGCGTGAAAT
GT
TCAGTACAGCAGAAGGATCAAGCTGGGAAAGTGATTGCGTCCATGATGGCTCATGATC

CC
GTCGCTTGGAAAGTTTTCCTCACCAGGTACGACGGAATCAAAAAAGTTCAAAGGATAC
TA
GGATAG
> comp10232_c0 (SlitCSP6)
GAGTGTTCCACTATACTCTTGGACAGGATTCTATTGTCTAGTGATCGGATTTTTAAACTT
ACTTACAGTATGAATTTCCTCGTGTTGTCGATTGTGGTTACAATGGCGGCCTTCGTAGCT
GCTGAAACTTATACGGACAGATACGATCACATCAACATCGACGAGATCATTGAGAACA
GG
AAACTGCTGGTGCCTTACATTAAGTGTACCCTGGACCAAGGAAGATGTACTCCAGAAG
GC
AGGGAACTGAAAGCTCATATCAAGGACGCTATGCAGACCTCCTGCTCCAAATGTACTG
AG
AAACAGAAGAAGGGTGCCAGGAAGGTCGTCAGACACATCAGGGCCAAGGAACAGGA
GTAC
TGGAAGCAGATCCTCGCCAAGTACGACCCTGAAGACCAATACAAAGAGAACTACGAA
ACC
TTCCTCGCTGCTGAAGACTAA
> comp19007_c0 (SlitCSP9)
TTTAAACAATCCTTCACCATGAAAGTCGTGTTCCTCGTGTGTGTGTTGGCTGCAGTGGT
G
TACGCCCACCCTCATGAGTCCCATTACACGGACAAATGGGACAACATCGACCTGGACG
AG
ATCCTAAACAACAAGAAAATCCTGGCTTCTTATGTGAAATGCTGCCTGGACCAGGGCA
AG
TGTACACCTGATGCCAAGGAATTGAAGTCGCACATTAAGGAAGCTTTGGAAAATCGAT
GT
GGTAAATGTACCCCAGCTCAGAAGGATGGTACTCGCAAGGTGTTGACACACCTCATCA
AC
CACGAGCCAGAAATGTGGAACCAGCTCTGCGAGAAATACGACGCCGAAGGCAAATAC
AGA
AAAATGTATGAAGACGAATACAAATCCGTTAAACACTAA
> comp16603_c0 (SlitCSP8)
AACGCACTCCATCTCACCACTGAACAAACCTCTCATACAAACAGAATCAAAATGAAGT
CC
ATGATCGTGCTCTGCGTGCTGTCGGTGGCGGCGCTGGTGGTGGCGCGGCCCGACGACT
CC
CACTACACCGACCGCTACGACAACGTCAACCTGGACGAAATCCTCTCCAACCGCCGCC
TG
CTGGTGCCCTACGTCAAGTGCATCCTCGACCAGGGCAAGTGCGCCCCTGACGCCAAGG
AG
CTAAAGGAACACATCAGGGAAGCCCTGGAGAACGAATGTGGCAAATGTACTGAGACC
CAG
AAGAACGGAACCCGGCGTGTGATCGAGTACTTGATCAACAACGAGGAGGAGTACTGG

AAC
GAGCTCACGGTCAAGTACGACCCTGAGAGGAAGTACACCGCCAAGTACGAGAAGGAA
CTC
AAGAAGATCAAGGCTTAA
> comp13797_c3 (SlitCSP5)
GCTGTTGGGCAGTACTCCTCAGGAAAACACGATCAAAGAATGAAATCCTTCATAGTTT
TG
TGTTTGTTCGGTCTGGCTGCGGTGGCTCTGGCCAAGCCTAATGGCAGCACTTACACTG
AC
AGATACGACAACGTCAATCTGGATGAGATCCTTGGTAATCGTCGTCTCCTGACGCCTTA
C
ATTAAGTGCATCCTCGAAGAGGGCAAGTGCACTGCTGATGGCAAGGAACTGAAATCTC
AC
ATCAGGGAGGCTTTAGAACAGAACTGTGCCAAATGTACCGATGCTCAGCGCAGTGGAA
CC
CGTAGGGTCTTGGGCCACATCATCAACAATGAGGAGGAATCCTGGAACCGTCTCAAGG
CT
AAATATGACCCCGAGTCCAAATACACTGTCAAATACGAACTGGAACTCAGAAAACTGA
AG
CAATAA
> comp19433_c0 (SlitCSP1)
GTCACTTGGCTACTTGAGATTACCGTACACATCAGGAACAAAACGGACAACATGAAAT
TC
GTACTAGTATTGTGCCTGATGACTGCCGCAGTCTTGGCTGAAGATGAAAAGTACCCTAG
C
AAGTACGACAACATCGACCTGGATGAGATCCTTGCCAACAAGAGGCTCCTCACTGCCT
AC
GTCAACTGCATCATGGAGAGAGGAAAGTGCAGTCCTGAGGGCAAGGAACTGAAAGA
ACAT
CTGGTAGACGCCATTGAGACTGGATGCTCGAAATGTACTGAAGCTCAAGAAAAGGGA
GCA
TACAAGGTCATCGAACACCTGATCCAGAACGAGCTGGACACCTGGCATGAGCTGACCG
AC
AAGTACGACTCCTCTGGCAAGTGGAGGAAGACATACGAAGACCGCGCCAGGGCTAAC
GGC
ATCGTCATCCCAGAATAA
> comp19885_c0 (SlitCSP14)
GATTGCGAATTCATGGTCACAGCCCAGCGAAGGGTCGAACGGAGCTCACGGGAAATA
CGA
TACAAAATGAAACTAATCATCGTACTCGCTTTATGTGTGGTGGCAGTGGCTTGGGCTAG
G
CCTGCCTCCACCTACACCGATAAGTGGGATAACATCAACGTAGACGAGATCTTAGAGTC
C
CAGCGTCTACTAAAAGCGTACGTAGACTGCTTGCTCGACAGAGGACGTTGCACTCCTG

AT
GGGAAAGCCCTCAAAGAAACTCTCCCTGACGCCTTAGAACATGAGTGTAGCAAATGTA
CT
GAAAAACAAAAGAAGAGCTCGGACAAGGTCATCAGGCACTTGGTGAATAAACGCCCA
GAC
TTGTGGCAGGAGTTATCAGGAAAGTATGACCCTGAGAATATCTACCAGGAGAGGTACA
AA
ACCCAGCTTGACGCAGTAAAGCGACATTAA
> comp24113_c0 (SlitCSP4)
TTGAACGAAGCTGTATTTTCTTCAGCTATTCTTGGAATGATGAAGAAGTCATTTATAACA
ATGATGCTTCTAATATACTTGACCATTCAGAGCAATGCTACGGAGACATCCACGTATACG
ACAAAGTATGATGGAATTGATCTGGATGAGATACTTAGCAACGACCGTCTGCTGACTGG
G
TATGTCAACTGTCTGATGGATCTTGGACCTTGCACGGCTGATGGAAAAGAATTAAAAA
AG
AACCTTCCGGATGCTATAGAGAATGATTGTAAGAAGTGTACTGAGCGACAGCGTGAGG
GT
GCTGACCGTGTCTGTCATTACCTCATCGACAATAGACCCGAAGAATGGACTAAACTCG
AG
GAGAAATACAAATCGGATGGAAGTTACAGAGCTAAATATTTAGCTAGCAAGGAAGCCA
CA
GATGAGAAGGCATCAAATGTCACAAACTCCAATGAAGATACAAATAATGTTTCTAAAG
AA
TAA
> comp19885_c0 (SlitCSP15)
CAAACAAGGCTTTATATACAACTAGGTATTTCACTTCCTCGGCAGTTGTGTTTTTCTCAC
CAGGCTGGACATATTTCACAGTTCATCATGAAGGCTGACTGTTTGTTAATTATGACATTA
ATGGCAGTGGTGGCAGCCGACTTCTACAACTCCAAGTACGACAGTTTTGATGTCCAGC
CC
TTACTGGAGAATGATAGGATACTACTGAGCTACACTAAATGTTTCCTAGACCAAGGACC
T
TGTACTCCTGACGCCAAAGATTTCAAAAAAATAATACCTGAAGCTCTAGAAACTACGT
GT
GGTAAATGCAGTCCAAAGCAGAAACAATTAATAAAGAAAGTAATCAAGGCTGTGATAG
CT
AAACACCCTGAGGCTTGGGAACAACTCAGTGATAAATACGACAAGGACAAAAAGTAT
AAA
GACTCCTTCGACAAATTCTTAGCCGAAAAAGAATAA
> comp20083_c0 (SlitCSP 17)
ACGCCGCAAGAGGAGCACTTGCACAACTTTCTTTCGGTAACCAATAACATGAGGAGCT
GG
CTGTTATGTCTGTGTGTGCTGACGGTGGTGGTGTCATGCTACTCCCAAGCCAATCGCTA
C
GAGAACTTCAACCCTGATGCCATCGTACAAAACGACAGGATTCTGCTAGCTTACTACA

AG
TGCGTGATGGACAAAGGACCTTGCACGAGAGACGGCAAGAATTTCAAACGTGTTTTAC
CA
GAGACGCTGGCCACTGCGTGCGGGCGCTGCAACCCTAAACAGAAGACGATCGTGCGC
AAG
CTTCTCCTGGGCATCAGGTCCAAGAGCGAACCTCGTTTCCTAGAACTCCTGGACAAAT
AC
AACCCTGACCGCTCCAACAGAGATGCTTTATACGCTTTCCTTGTAACCGGTGCTTAA
> comp20502_c0 (SlitCSP13)
CATCCTATTACAGTATCACGCATCATCATGAAACTAGTAATCATCTTGGCGTTGGTGGCA
GTAGCCCTGGCCCGCCCTGATGACGGTGGCTTCTACGACAAGAAGTACGATAGCTTCA
AC
GCTGATGAGCTCATTGAAAATGAACGTTTGCTGAAATCGTACGCGCACTGTTTCCTCGG
T
GATGGCAAGTGCACTCCAGAAGGAAACGATTTCAAGAAATGGATCCCTGAAGCCACA
ACG
ACATCTTGTGGGAAGTGCACGGAAAAACAAAAGGCTCTCATTGCAAAAACTATCAAG
GCG
ATCAAGGACAAACTTCCAACAGAATATGAAGCTCTAATAAAGAAGCACGACCCTGAAA
AT
AAACACCATGACGACCTCGACAAGTTCCTACAGAAATACTCTCATTAG
> comp20836_c0 (SlitCSP11)
GAAGGGATATCTGAAAATCTAAACATGAAGAGCATCCTAGTTCTGTGCTTGTTGGTCGC
G
GCTGTCTCCTGCAGACCAGAGAGTTACGACACTAGATATGACAACTTTGATGTTGAAG
CC
TTGGTCGGCAACGTTCGACTGCTGACTGCGTACGGTCACTGCTTCCTTGGCAATGGAC
CT
TGCACTCCAGAGGGAAGTGCTTTTAAAAAAACCATCCCCGACGCTCTTAGGACCGGCT
GT
GGCAAATGTTCACCGAAACAGCGTCACTTGATCCGTGTCGTTGTACAAGGCTTCCAAA
AT
AAGACCCCAGCCCTTTGGCAGGACCTGGTCAAGAAGCAGGACCCCAACGGACAGTAC
AAA
GAAATCTTCACCAGGTTCTTGAACGGCAGAGACTAA
> comp19065_c0 (SlitCSP3)
AAGAAATCAACTGTGAAAATGAGGGTTCTGGTTGTACTGTCGTGCTTGGTCGTGGTGG
TT
TTCGCTGCTGATAAATACAACCCTAAATATGATAACTTTGACGTGGAGACCCTGATCTCC
AACGACAGATTGCTGAAGGCATACATCAACTGCTTCTTGGAAAAAGGACGTTGCACGC
CC
GAAGGATCAGATTTTAAAAAGGCCCTCCCCGAGGCGATAGAAACTACTTGCGCGAAGT
GC
ACAGACAAACAGAAGGGTAACATCAGAAAGGTGATCAAGGCCATCCAGCAGAAACAC

CCC
AAGGAGTGGGAAGACCTCGTCAAGAAGAATGACCCCAGCGGCAAACACCGCGGTAA
CTTC
GACAAATTCATCCAGGGAAGCAGCTAA
> comp40932_c0 (SlitCSP18)
TTGGGACATCAGATCACACGAATCGCAATAAAATTCAAAATGAACGCGCTACTGATAGC
T
GTCTTCGCCTTAGCTGCTCCACTTGCCTTTGGCTATGACGAAAAATATGACAAATTAGA
C
GTCGACAAGATTCTGGGAGATGATGCACTTTTCACCGCATACATTAATTGCATGTTGGA
T
AAGGGACCCTGCTCTGTAGAACATTCTGCTGATTTCAGACAATTGCTGCCCGAAGTGAT
A
GCCACAGCTTGCGAGAAATGCACTCCCATACAGAGGCAAAATGTCAGGAAGACAGTC
AAG
GCTCTCAGTGAGAAGAAACCTGATGACTTCGTCCAATTCCGAGCCAAATTCGATCCTA
AA
GGCGAATACGAAAAGGCCTTCAGTGCTTTCGTCATCGGTACCGACTAA
> comp203796_c0 (SlitCSP19)
ATTGCAGTAATGAACGGTGCAAGTAAATGCAACTTGTTAATTTTTATGTTTCTATTTGTT
GCAACTGTTGTTTCGCAAGAGAAGTTCTATGATAGAAGATACGATTATTACGAAATAGA
C
ACTTTGATACAGAATCCTAGGTTACTGAAGAAGTATTTGGATTGTTTTCTTTGGAAAGG
A
CCCTGTACACCCATAGGAAGAGTGTTCAGACAAATCCTGCCCGAAGCGGTTCAGACAG
CG
TGTAAGAAGTGCACTCCATCACAGCGGCGACTGGCAAGAAAAACGTTCAACGCTTTC
AAG
GGATATTTCCCAGAGATACATGAGGAACTGAGGAAGAAACTTGACCCAAAAAACAAGT
AC
TATGAA
> comp19506_c0 (SlitCSP12.2)
GCATTATTCGTCCACAACTCTTTATTCTTAAATAGGAATATCAATCCTGACACGATGAAG
TGCATCTACGTGTTATCTGTTCTGCTGGTTTTTGCTGCTGTCCAGGCTGACGAGAAGTA
C
AGCTCGGAGAATGATGACCTGGACATCGACGCCGTGGTAGCTGACGTTGATGCCCTCA
AA
GGCTTTGTTGGGTGCTTCATGGACGCTGTGACTTGCCACGCTGTTGCTGCCGATTTCAA
A
AAGGATCTTCCTGAAGCGGTAGCGACAAGCTGCTCTAAATGTACGGATGCCCAGAAGC
AT
ATCTTCCACAGGTTCCTTCTCGGACTGAAACAGAAGCTGCCTGCTGACTACGAAGCCT
TC
AAGAAGAAGTTTGACCCTGAAGGTCTACACTTCCATACTCTTGAGGCCAATGTTGCCA

AC
TCTTAA
> comp22263_c0 (SlitCSP12.1)
CTAGACTATCAATCGTTAGACCGTTTTGGTTTTAGTCTGCATCAATCCAGTTCGGGACG
C
GTCCTCTTCAATAGAAGCAAAATGAAGTACATATTAGTGGCTCTCGTTGTAACCATAGC
G
GTGGTGGACGCTCAAGAAACGTATGGGACCGAATATGATAATGTAAATGGAGAAGCTA
TC
GTTTCTGATGACAAACAGTTCCAAGGATTTGTCGACTGTTTCACGGGCGCTGCTCCTTG
C
AACGAGCCTGCTGCTGCTTTCAAAAGGGTTTTACCTGAAGCGATTGTACAGGCTTGTG
GC
AAATGTAATCCAGCACAAAAGCACTTAGTAAGGCTTTTCTTAGAAGCCTACTCGAAGA
AA
AGGCCACAAGAGTACGAAAAGTTCAAAGATTTATTTGATCCTGAAAGGAAATACTTTC
CC
AAATTCGAAGCATCCGTTGCCGGGTTTTAA
